# Urinary Surgery

**Published:** 1911-06

**Authors:** 


**Urinary Surgery.** By Frank Kidd, M.B., B.C., F.R.C.S. Pp. xvi., 429. London: Longmans, Green & Co. 1910.

This book is an excellent review of urinary surgery, surveying almost completely all the modern methods and advances made in this subject, particularly in the direction of the estimation of the functional capacity of the kidneys by means of ureteral catheterisation, in the bacteriology of the urine, and in the value of the X-rays and the development of the clinical interpretation of their shadows.

The classifications are good, and etiology, pathology, the most important signs and symptoms, and diagnosis, prognosis and treatment are dealt with in a concise and orderly manner.

It co-ordinates the many advances made in urinary surgery which has converted it from a groping in the dark into almost an exact science, and will therefore be found a most useful book from the general practitioner's point of view, and for those entering for the higher surgical examinations.

